# 淋巴结外周T细胞淋巴瘤诊断与治疗中国专家共识（2025年版）

**DOI:** 10.3760/cma.j.cn121090-20250808-00364

**Published:** 2025-12

**Authors:** 中国临床肿瘤学会（CSCO）淋巴瘤专家委员会 中华医学会血液学分会淋巴细胞疾病学组

## Abstract

外周T细胞淋巴瘤（peripheral T-cell lymphomas, PTCL）是我国常见的非霍奇金淋巴瘤，发病率高于西方国家。除结外NK/T细胞淋巴瘤外，我国最常见的PTCL是淋巴结PTCL。淋巴结PTCL多数侵袭性强、预后差，各亚型分子特征和临床表现差异大，传统化疗疗效有限。近年来靶向药物以及多药联合治疗策略的应用显著改善了部分患者的预后，也正在改变患者的初始、维持及复发后治疗模式。基于此，中国临床肿瘤学会（CSCO）淋巴瘤专家委员会和中华医学会血液学分会淋巴细胞疾病学组组织相关专家制定了本共识，旨在推动我国临床医师对淋巴结PTCL的进一步规范诊疗及新型疗法的临床研究和应用。

## Abstract (en)

Peripheral T-cell lymphomas（PTCL）are a common type of non-Hodgkin lymphoma in China, with a higher incidence compared to Western countries. Apart from extranodal NK/T-cell lymphoma, nodal PTCL is the most prevalent subtype in China. Nodal PTCL is highly aggressive with a poor prognosis. Significant differences in molecular characteristics and clinical manifestations are observed among various subtypes, and the efficacy of conventional chemotherapy is limited. In recent years, the application of targeted therapies has significantly improved outcomes for some patients and is changing treatment paradigms for initial, maintenance, and relapsed disease. Based on these advancements, the Lymphoma Expert Committee of Chinese Society of Clinical Oncology and Lymphoid Disease Group, Chinese Society of Hematology, Chinese Medical Association have organized a panel of experts to develop this consensus, aiming to promote standardized diagnosis and treatment of nodal PTCL among clinicians in China, as well as to facilitate clinical research and application of novel therapies.

外周T细胞淋巴瘤（peripheral T-cell lymphomas, PTCL）是一组来源于成熟T细胞和NK细胞的具有高度异质性的非霍奇金淋巴瘤（NHL）。流行病学数据显示，PTCL的发病率存在显著的地域差异，在亚洲国家中PTCL占NHL的15％～25％，在我国PTCL占NHL的25％～30％，高于西方国家的10％～15％[1]–[3]。2022年WHO第5版淋巴造血系统肿瘤分类标准将T细胞和NK细胞肿瘤归于一个大类，包含9类34种亚型，强调分子遗传学特征在诊断和预后中的重要性[4]。根据发病部位，PTCL分为淋巴结PTCL、结外PTCL、白血病性PTCL和原发皮肤T细胞淋巴瘤[5]。

除结外NK/T细胞淋巴瘤（natural killer/T-cell lymphoma, NKTCL）外，我国PTCL最常见的亚型是淋巴结PTCL，包括PTCL⁃非特指型（NOS）、淋巴结滤泡辅助T细胞淋巴瘤（nodal T-follicular helper cell lymphoma, nTFHL）和间变性大细胞淋巴瘤（anaplastic large cell lymphoma, ALCL）。nTFHL包括“nTFHL，血管免疫母细胞型（nTFHL-AI）”、“nTFHL，滤泡型（nTFHL-F）”和“nTFHL-NOS”三个亚型。

除间变性淋巴瘤激酶（anaplastic lymphomakinase, ALK）阳性ALCL预后较好外，其余大部分淋巴结PTCL类型预后不佳[5]–[6]。近年来，针对PTCL的新型靶向药物不断研发与创新，并越来越多地应用于临床，显著改善了部分患者的预后，也正在改变患者的初始、维持及复发后治疗模式[7]–[8]。基于此，中国临床肿瘤学会（CSCO）淋巴瘤专家委员会和中华医学会血液学分会淋巴细胞疾病学组组织多学科专家，系统梳理国内外指南共识和研究进展[5],[9]–[10]，特别是近年来国内相关临床研究成果，经充分讨论形成针对淋巴结PTCL诊疗的专家共识，旨在推动我国淋巴结PTCL的规范诊疗及新疗法的临床研究和应用。

一、流行病学

我国PTCL的主要类型包括PTCL-NOS、血管免疫母细胞性T细胞淋巴瘤（angioimmunoblastic T-cell lymphoma, AITL）、ALK^+^ALCL和ALK^-^ALCL。国内多中心10 002例患者的病理分析显示，上述亚型分别占所有淋巴瘤的4.25％、2.66％、1.57％和0.90％，占T细胞和NK细胞淋巴瘤的19.88％、12.44％、7.34％和4.21％[11]。

二、诊断及鉴别诊断、分期和风险评估

（一）PTCL的诊断与鉴别诊断

PTCL的精准诊断依赖于肿瘤组织活检的病理诊断，包括细胞形态学、免疫组化、流式细胞术及分子生物学检查等多种检测技术。在不能获得组织活检的情况下粗针穿刺可以替代，但可能影响诊断和分型，不建议细针穿刺[9]–[10],[12]。免疫组化标记应包含B细胞标记（CD10、CD19、CD20、CD79a、PAX5、CD22、BCL6、MUM1/IRF4、CD38, CD138、BCL2），T细胞标记（CD3、CD2、CD5、CD7、CD4、CD8），T细胞功能亚型标记（CD25、FoxP3、PD-1/CD279、CXCL13），细胞毒性T细胞标记（TIA-1、Granzyme B、Perforin），T细胞发育阶段标记（CD1a、CD99、TdT）以及CD30、ALK、CD56、TCRβ、TCRδ、Ki-67和EB病毒编码的小RNA（Epstein-Barr virus-encoded small RNA，EBER）原位杂交。

分子生物学检测有助于PTCL亚型的精准诊断，推荐有条件的单位开展。通过基因表达谱分析可将PTCL-NOS分为PTCL-TBX21和PTCL-GATA3亚型，TBX21亚型的免疫组化为TBX21^+^CXCR3^+^，GATA3亚型的免疫组化为GATA3^+^CCR4^+^。PTCL-TBX21亚型的预后优于PTCL-GATA3亚型，5年总生存（OS）率分别为38％和19％。nTFHL的分子特征主要涉及表观遗传学调控基因TET2、DNMT3A、IDH2^R172K^和RHOA^G17Val^突变。ALK^+^ALCL的细胞遗传学和分子生物学特点是染色体易位t（2;5），TP53、EP300、KMT2D、TCR和NOTCH1信号通路基因突变；ALK^-^ ALCL需检测TP63、DUSP22/IRF4及JAK2重排。

PTCL的鉴别诊断主要包括T淋巴母细胞白血病/淋巴瘤、霍奇金淋巴瘤、B细胞淋巴瘤和反应性淋巴结增生，还需要和惰性克隆性T细胞或NK细胞增殖性疾病鉴别。

（二）PTCL的分期和预后评估

PTCL分期主要参考2014版Lugano分期标准（表1），分为局限期（Ⅰ期、Ⅱ期）和进展期（Ⅲ期、Ⅳ期）。淋巴结PTCL往往也有结外部位累及，分期检查首先推荐PET-CT，其次是应用CT检查颈部、胸部、腹部和骨盆。MRI有助于诊断中枢神经系统累及。由于PET-CT对诊断骨髓累及敏感性低，需要骨髓穿刺和活检。

**表1:** 2014版Lugano分期标准

分期	描述
局限期	
Ⅰ期	仅侵及单一淋巴结区域（Ⅰ），或侵及单一结外器官不伴有淋巴结受累（ⅠE）
Ⅱ期	侵及≥2个淋巴结区域，但均在膈肌同侧（Ⅱ），可伴有同侧淋巴结引流区域的局限性结外器官受累（ⅡE）（如甲状腺受累伴颈部淋巴结受累或纵隔淋巴结受累直接延伸至肺受累）
Ⅱ期伴大包块^a^	Ⅱ期伴有大包块
进展期	
Ⅲ期	侵及膈肌上下淋巴结区域（Ⅲ），或侵及膈上淋巴结+脾受累（ⅢS）
Ⅳ期	侵及淋巴结引流区域之外的结外器官（Ⅳ）

**注** 根据2014年Lugano标准，不再对淋巴瘤的大包块（bulky）病灶进行具体的数据限定，只需在病例中明确记录最大病灶的最大径即可；Ⅱ期伴有大肿块的患者，应根据病理类型及疾病不良预后因素酌情选择治疗原则，如伴有大包块的惰性淋巴瘤患者可选择局限期治疗模式，但伴有大包块的侵袭性淋巴瘤患者应选择进展期治疗模式

PTCL的预后分层主要为国际预后指数（international prognostic index, IPI），适合所有PTCL患者，其纳入指标包括年龄、血清乳酸脱氢酶、体能状态、疾病分期、结外部位受累数目。低危（0～1分）、低中危（2分）、中高危（3分）和高危（4～5分）组5年生存率分别为73％、51％、43％和26％[13]。年龄调整的IPI（aaIPI）适用于年龄≤60岁的患者。IPI和aaIPI依据1993年发布的前瞻性临床研究结果制定[13]。现阶段分子分型可能有助于临床更精准地判断预后[12]。因此，推荐采用IPI评分，有条件的单位建议开展分子分型。T细胞淋巴瘤的预测指数（prognostic index in T-cell lymphomas, PIT）纳入了年龄、体能状态、乳酸脱氢酶及骨髓侵犯，适用于PTCL-NOS，但其依据回顾性临床研究结果制定。2018年，国际T细胞淋巴瘤项目制定了一种新的预后评分标准T-CELL SCORE，适用于PTCL-NOS，主要特点是纳入血清白蛋白、中性粒细胞绝对计数、分期和体能状态作为评分标准[14]。

三、治疗

本共识以两部分阐述淋巴结PTCL的治疗，治疗流程见图1。

**图1:**
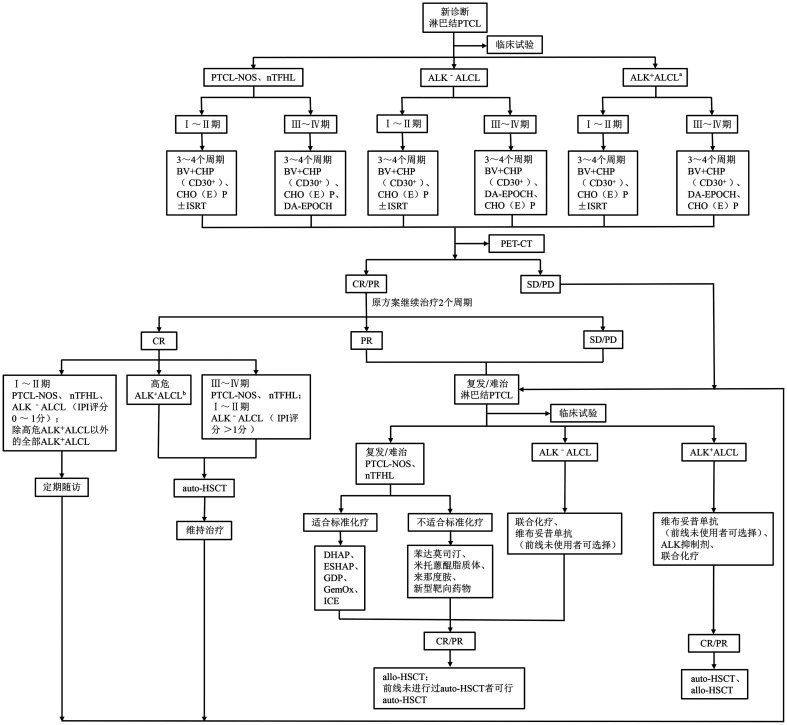
淋巴结PTCL治疗流程图 **注** PTCL：外周T细胞淋巴瘤；PTCL-NOS：外周T细胞淋巴瘤非特指型；nTFHL：淋巴结滤泡辅助T细胞淋巴瘤；ALK^+^ALCL：间变性淋巴瘤激酶阳性的间变性大细胞淋巴瘤；ALK^-^ ALCL：间变性淋巴瘤激酶阴性的间变性大细胞淋巴瘤；auto-HSCT：自体造血干细胞移植；BV+CHP：维布妥昔单抗+环磷酰胺+阿霉素+泼尼松；CHO（E）P：环磷酰胺+阿霉素+长春新碱（+依托泊苷）+泼尼松；ISRT：受累部位照射；DA-EPOCH：剂量调整的依托泊苷+长春新碱+阿霉素+环磷酰胺+泼尼松；PET-CT：正电子发射计算机断层显像；CR：完全缓解；PR：部分缓解；SD：疾病稳定；PD：疾病进展；DHAP：地塞米松+阿糖胞苷+顺铂；ESHAP：依托泊苷+甲泼尼龙+阿糖胞苷+顺铂；GDP：吉西他滨+地塞米松+顺铂；GemOx：吉西他滨+奥沙利铂；ICE：异环磷酰胺+卡铂+依托泊苷；allo-HSCT：异基因造血干细胞移植。^a^ALK^+^患者NCCN指南不推荐首先参与临床试验；^b^高危ALK^+^ALCL：肿瘤负荷高，IPI评分>2分，年龄>40岁，3个周期化疗后未达CR

（一）新诊断淋巴结PTCL的治疗

1. 诱导治疗：根据国内外指南，淋巴结PTCL的一线治疗推荐以蒽环类药物为主的联合化疗，共6个疗程。化疗方案包括CHOP（环磷酰胺+阿霉素+长春新碱+泼尼松）、CHOEP（环磷酰胺+阿霉素+长春新碱+依托泊苷+泼尼松）、DA-EPOCH（剂量调整的依托泊苷+长春新碱+阿霉素+环磷酰胺+泼尼松）等。CHOEP方案能使IPI评分<1分的PTCL-NOS、AITL和ALK^-^ ALCL患者无事件生存获益[5]。在化疗时代，除ALK^+^ALCL外，其他PTCL亚型的一线治疗疗效普遍不理想，远期生存率低[15]–[16]。因此，参加临床试验也作为一线推荐。ALK^+^ALCL患者优先推荐维布妥昔单抗（BV）+CHP（环磷酰胺+阿霉素+泼尼松），该方案也可用于CD30阳性（CD30表达率≥10％）的其他PTCL亚型，但疗效优势尚无循证证据[17]–[18]。3～4个疗程结束时的中期评估十分重要，中期评估为无治疗反应或疾病进展者按复发/难治（relapse/refractory，R/R）PTCL治疗；评估疗效为部分缓解（PR）以上者继续按原方案完成2个疗程后再进行评估，无法达到完全缓解（CR）患者均按R/R PTCL治疗。

对于ALK^+^ALCL的Ⅰ/Ⅱ期患者，诱导治疗阶段可酌情选择4～6个疗程联合化疗，必要时结合受累部位照射；而对于PTCL-NOS、nTFHL以及ALK^-^ ALCL的Ⅰ～Ⅳ期患者，诱导治疗阶段仍首先推荐以6个疗程联合化疗为主，必要时结合受累部位照射的治疗策略。

新药或小分子靶向药物联合化疗是一线治疗的新策略，特别是针对不同分子特点的患者。复旦大学附属肿瘤医院的前瞻性单臂临床研究采用含聚乙二醇脂质体阿霉素的CHOP方案一线治疗PTCL，CR率为62.5％，5年和8年无进展生存（PFS）率分别达到55.1％和52.0％，5年和8年OS率分别达到62.5％和54.3％，心脏不良反应明显减少[19]。中山大学肿瘤防治中心的回顾性研究显示，与单纯化疗相比，化疗联合西达本胺治疗可以使IPI高危患者的PFS获益，但对OS无明显影响[20]。该中心的另一项前瞻性多中心临床研究，采用CMOP（米托蒽醌脂质体+环磷酰胺+长春新碱+泼尼松）方案一线治疗PTCL患者，同样显示出有效的抗肿瘤活性，客观缓解率（objective response rate, ORR）和CR率分别为84.0％和52.0％[21]。上海交通大学医学院附属瑞金医院牵头了一项多中心Ⅱ期非随机临床研究（GUIDANCE-03），在CHOP方案基础上联合靶向药物（CHOP-X方案），从第2个疗程开始对p53突变患者加用地西他滨，TET2/KMT2D突变患者加用阿扎胞苷，CREBBP/EP300突变患者加用西达本胺，无上述突变患者加用来那度胺。CHOP-X方案和CHOP方案的CR率分别为64.6％和33.3％（*P*＝0.004），中位PFS期分别为25.5个月和9.0个月（*P*＝0.041），但中位OS期相似[22]。苏州大学附属第一医院和四川大学附属华西医院的两项回顾性临床研究均显示，西达本胺联合CHOP方案治疗组的疗效和生存较CHOP方案组有优势[23]–[24]。青岛大学附属医院开展了一项CPET（西达本胺+依托泊苷+沙利度胺+泼尼松）方案治疗初治AITL患者的前瞻性多中心Ⅱ期临床研究，入组患者中年龄≥60岁者占34.6％，ORR和CR率分别为90.2％和54.9％，中位PFS期达42.6个月，最常见的3～4级不良反应是粒细胞缺乏（32.3％），提示该方案更适合老年患者[25]。天津医科大学肿瘤医院探索了CMOEP方案在初治PTCL中的治疗前景，ORR和CR率分别为100％和66.7％，且安全性可控[26]。上述研究的疗效总结见表2。

**表2:** 国内PTCL一线应用含新药或小分子靶向药物联合化疗方案疗效

研究	牵头单位	例数	方案（治疗组/对照组）	PTCL-NOS［例数（％）］	AITL［例数（％）］	ALCL［例数（％）］	ORR（治疗组/对照组）	CR率（治疗组/对照组）
ChiCTR2100054588（Ⅱ期）[19]	复旦大学附属肿瘤医院	40	聚乙二醇脂质体阿霉素+COP方案	19（47.5）	9（22.5）	11（27.5）	82.5％	62.5％
回顾性研究[20]	中山大学附属肿瘤医院	104	西达本胺+化疗/化疗	47（45.2）	26（25.0）	NA	77.4％/72.6％（*P*=0.608）	54.8％/47.9％（*P*=0.520）
GUIDANCE-03（Ⅱ期）[22]	上海交通大学医学院附属瑞金医院	96	靶向药物+CHOP/CHOP方案	18（19.0）	61（64.0）	7（7.0）	66.7％/52.1％（*P*=0.212）	64.6％/33.3％（*P*=0.004）
NCT05458180（Ⅰ期）[26]	天津医科大学肿瘤医院	13	CMOEP方案	4（30.8）	3（23.1）	3（23.1）	100％	66.7％
NCT04548700（Ⅰ期）[21]	中山大学肿瘤防治中心	26	CMOP方案	4（15.4）	14（53.8）	4（15.4）	84.0％	52.0％
回顾性研究[23]	苏州大学附属第一医院	66	西达本胺+CHOP/CHOP方案	NA	66（100）	NA	81.8％/63.6％（*P*=0.034）	72.7％/42.4％（*P*=0.002）
回顾性研究[24]	四川大学附属华西医院	86	西达本胺+化疗/化疗	NA	86（100）	NA	84.3％/60.0％（*P*=0.011）	60.8％/42.9％（*P*=0.102）
NCT03273452（Ⅱ期）[25]	青岛大学附属医院	71	CPET方案	NA	71（100）	NA	90.2％	54.9％

**Table:** 

研究	中位DOR（月）（治疗组/对照组）	中位PFS期（月）（治疗组/对照组）	中位OS期（月）（治疗组/对照组）
ChiCTR2100054588（Ⅱ期）[19]	未达到，2年、5年、8年持续缓解率分别为58.7％、55.8％、55.8％	未达到，5年、8年PFS率分别为55.1％、52.0％	未达到，2年、5年、8年OS率分别为80.0％、62.5％、54.3％
回顾性研究[20]	14.0/10.0（*P*=0.135）	12.4/8.5（*P*=0.047）	17.1/16.5（*P*=0.212）
GUIDANCE-03（Ⅱ期）[22]	NA	25.5/9.0（*P*=0.041）	未达到/30.9（*P*=0.088）
NCT05458180（Ⅰ期）[26]	未达到	未达到，6个月、1年PFS率分别为91.7％、53.5％	未达到，1年、18个月OS率分别为91.7％、61.1％
NCT04548700（Ⅰ期）[21]	6.1	8.8	未报道
回顾性研究[23]	NA	22.0/11.0（*P*=0.080）	未达到/20.0（*P*=0.002）
回顾性研究[24]	未达到	27.0/12.0（*P*=0.025）	未达到/48.0（*P*=0.225）
NCT03273452（Ⅱ期）[25]	24.7	42.6	未达到

**注** PTCL：外周T细胞淋巴瘤；PTCL-NOS：外周T细胞淋巴瘤，非特指型；AITL：血管免疫母细胞性T细胞淋巴瘤；ALCL：间变性大细胞淋巴瘤；ORR：客观缓解率；CR：完全缓解；DOR：缓解持续时间；PFS：无进展生存；OS：总生存；COP：环磷酰胺+长春新碱+泼尼松；CHOP：环磷酰胺+阿霉素+长春新碱+泼尼松；CMOEP：盐酸米托蒽醌脂质体+环磷酰胺+长春新碱+依托泊苷+泼尼松；CMOP：盐酸米托蒽醌脂质体+环磷酰胺+长春新碱+泼尼松；CPET：西达本胺+泼尼松+依托泊苷+沙利度胺；NA：无数据

2. 巩固治疗：对于Ⅲ/Ⅳ期PTCL-NOS、nTFHL、ALK^-^ ALCL以及高危（IPI评分≥3分）ALK^+^ALCL患者，推荐在首次缓解后4～6个月内进行自体造血干细胞移植（auto-HSCT）[5],[27]–[28]。国际前瞻性研究结果显示，淋巴结PTCL首次缓解后接受auto-HSCT，中高危患者和AITL亚型有生存获益[29]。auto-HSCT巩固治疗不适合体能状态差（KPS评分<50分或ECOG评分≥3分）、年龄≥65岁及器官功能障碍或活动性感染患者。国际前瞻性随机对照试验显示，年龄<60岁的PTCL患者接受异基因造血干细胞移植（allo-HSCT）或auto-HSCT后生存无差异[30]。解放军总医院报道的国内真实世界研究显示，低危患者（低PIT评分）和疾病控制良好者接受auto-HSCT较allo-HSCT有更多的生存获益[31]。中国医学科学院血液病医院牵头的一项多中心真实世界研究发现，对于淋巴结PTCL，一线治疗有效者进行allo-HSCT较auto-HSCT能获得更长的PFS期，复发率更低，非复发死亡率相似[32]。Ⅰ/Ⅱ期PTCL-NOS、nTFHL患者化疗3～4个疗程后可考虑累及野放疗（involved-site radiotherapy, ISRT）作为巩固治疗。无论AKL阳性还是阴性，非大肿块、IPI评分0～1分ALCL患者3～4个疗程联合化疗获得CR后也可考虑ISRT。ALK^-^ ALCL非大肿块但IPI评分>1分和ALK^+^ALCL高危患者接受6个疗程联合化疗后可考虑ISRT。

3. 维持治疗：PTCL具有侵袭性强、复发率高的特点，即使一线治疗达到CR，多数患者仍面临复发风险，预后较差[5]–[6]。在靶向药物时代，维持治疗对于延长缓解期、减少复发及改善OS可能具有重要价值。北京协和医院的一项前瞻性多中心Ⅱ期临床研究结果显示，一线治疗后接受auto-HSCT巩固治疗、西达本胺维持治疗或不维持治疗的2年OS率分别为85.6％、80.8％和69.0％，auto-HSCT巩固治疗组和西达本胺维持治疗组的PFS优于不维持治疗组[33]。郑州大学附属第一医院回顾性分析一线治疗获得CR或PR患者行auto-HSCT后接受西达本胺和沙利度胺维持或不维持治疗的疗效，两组患者的复发率分别为26.7％和52.2％，3年OS率分别为86.0％和54.2％，差异有统计学意义（*P*＝0.004）[34]。因此，诱导治疗获得PR的患者行auto-HSCT后建议接受维持治疗。国际一项多中心Ⅱ期研究显示，CD30^+^ PTCL患者接受BV-CHEP（维布妥昔单抗+环磷酰胺+阿霉素+泼尼松+依托泊苷）方案一线治疗6个周期，获得PR及以上患者行auto-HSCT后或在诱导治疗结束后接受BV维持治疗不超过10个周期，中位随访25个月，2年PFS率和OS率分别为59％和86％[35]。

一项国际小样本Ⅱ期研究显示，首次缓解行auto-HSCT出院后21 d内（干细胞输注后60 d内）接受帕博利珠单抗（PD-1单抗）维持治疗，18个月PFS率为83.6％，OS率为94.4％[36]。针对不适合移植的患者，吉林大学白求恩第一医院的一项回顾性研究初步提示，西达本胺一线巩固及序贯维持治疗可延长患者生存期并降低疾病进展风险。

（二）R/R淋巴结PTCL的治疗

R/R PTCL的定义参考NCCN临床实践指南：T细胞淋巴瘤（2025.V1）[9]。复发定义为治疗达到CR后再次出现相同疾病。难治定义为出现以下情况之一：①初治患者一线治疗方案应用3～4个周期后无反应或疾病进展；②初治患者一线治疗方案应用6个周期后未达到CR。

1. R/R PTCL的治疗方案：目前R/R PTCL患者无标准治疗方案，首先推荐参加临床试验。PTCL-NOS、nTFHL适合联合化疗者可以选择挽救化疗，主要包括DHAP方案（顺铂+阿糖胞苷+地塞米松）、ESHAP方案（依托泊苷+甲泼尼龙+顺铂+阿糖胞苷）、GDP方案（吉西他滨+地塞米松+顺铂）、GemOx方案（吉西他滨+奥沙利铂）和ICE方案（异环磷酰胺+卡铂+依托泊苷）。常用化疗方案的疗效差异见表3。上述多为回顾性研究或单臂Ⅱ期临床试验，普遍存在样本量小、入组患者临床特征异质性大（如未区分亚型及既往治疗线数）。缺乏大型随机对照临床试验。高强度化疗可能加重骨髓抑制及感染风险，导致部分患者因药物不良反应无法完成既定疗程[37]–[41]。PTCL-NOS、nTFHL不适合联合化疗者可以选择单药化疗如苯达莫司汀、米托蒽醌脂质体、来那度胺和新型靶向药物。

**表3:** 复发/难治外周T细胞淋巴瘤挽救化疗常用方案

化疗方案	完全缓解率	总生存（OS）
DHAP[22]	40％	2年OS率：36％
ESHAP[23]	39％	中位OS期：11.0个月
GDP[24]–[25]	48％	中位OS期：9.3个月（PTCL-NOS）
ICE[27]	40.0％（复发），27.3％（难治）	中位OS期：33.9个月（复发）；13.9个月（难治）

**注** DHAP：地塞米松+阿糖胞苷+顺铂；ESHAP：依托泊苷+甲泼尼龙+顺铂+阿糖胞苷；GDP：吉西他滨+顺铂+地塞米松；ICE：异环磷酰胺+卡铂+依托泊苷；PTCL-NOS：外周T细胞淋巴瘤非特指型

ALK^-^ ALCL的挽救治疗除化疗外，还应包括BV。ALK^+^ALCL患者首先推荐BV、ALK抑制剂，其次是联合化疗。

2. 新药和小分子靶向药物：近年来，新药和靶向药物的研发上市为R/R PTCL患者的治疗提供了更多治疗选择，已有多项单药方案获批用于R/R PTCL的治疗，如JAK抑制剂戈利昔替尼、组蛋白去乙酰化酶抑制剂（HDACI）西达本胺、叶酸代谢抑制剂普拉曲沙，以及针对CD30的抗体偶联药物（antibody-drug conjugate, ADC）BV等。本共识主要纳入国内已经上市的产品，这些药物靶向性强，单药或联合治疗越来越多地应用于临床（表4）。

**表4:** 新药和小分子靶向药物单药或联合治疗复发/难治PTCL的疗效

药物	临床研究项目名称	临床研究类型	例数	PTCL-NOS［例数（％）］	AITL［例数（％）］
单药					
戈利昔替尼[42]	JACKPOT8 Part B	单臂、国际多中心Ⅱ期研究	88	50（56.8）	16（18.2）
西达本胺[43]	ChiCTR-TNC-10000811	单臂、多中心关键Ⅱ期研究	79	27（34.2）	10（12.7）
米托蒽醌脂质体[44]	NCT03776279	单臂、开放标签、多中心Ⅱ期研究	97	32（33.0）	26（26.8）
普拉曲沙[45]	PROPELStudy	单臂、开放标签、国际多中心Ⅱ期研究	111	59（53.2）	13（11.7）
维布妥昔单抗[46]	NCT00866047	单臂、开放标签、多中心Ⅱ期研究	58	NA	NA
林普利塞[47]	ChiCTR20210333	单臂、开放标签、多中心Ⅱ期研究	88	24（27.3）	48（54.5）
联合治疗					
西达本胺联合泼尼松、环磷酰胺和沙利度胺[48]	NCT02879526	单臂多中心Ⅱ期研究	45	17（37.8）	20（44.4）
维布妥昔单抗联合ICE/罗米地辛联合ICE[49]	无	回顾性研究	13	6（46.2）	4（30.8）
克唑替尼联合化疗[50]	ChiCTR2000029373	单中心历史对照研究	47	NA	NA
罗米地辛联合普拉曲沙[51]	NCT01947140	单臂多中心Ⅱ期研究	14	6（42.9）	1（7.1）
靶向药物联合					
度维利塞联合罗米地辛[52]	NCT02783625	Ⅰb/Ⅱa期研究	98	26（26.5）	31（31.6）
西达本胺联合阿扎胞苷±GemOx[53]	ChiCTR2000037232	多中心Ⅱ期研究	30	4（13.3）	19（63.3）
罗米地辛联合阿扎胞苷[54]	NCT01998035	多中心Ⅱ期研究	25	4（16.0）	14（56.0）

**Table:** 

药物	ALCL［例数（％）］	ORR（％）	中位DOR（月）	中位PFS期（月）	中位OS期（月）
单药					
戈利昔替尼[42]	17（19.3）	44.3	20.7	5.6	19.4
西达本胺[43]	17（21.5）	28.0	9.9	2.1	21.4
米托蒽醌脂质体[44]	16（16.5）	46.3	6.8	8.5	23.3
普拉曲沙[45]	17（15.3）	29.0	10.1	3.5	14.5
维布妥昔单抗[46]	58（100）	86.0	25.6	20.0	未达到
林普利塞[47]	2（2.3）	48.0	未达到	5.5	14.2
联合治疗					
西达本胺联合泼尼松、环磷酰胺和沙利度胺[48]	2（4.4）	71.1	6.0	8.5	17.2
维布妥昔单抗联合ICE/罗米地辛联合ICE[49]	2（15.4）	66.7（维布妥昔单抗）；71.4（罗米地辛）	7.5（维布妥昔单抗）；6.0（罗米地辛）	NA	NA
克唑替尼联合化疗[50]	47（100）	65.0	55.4	未达到	未达到
罗米地辛联合普拉曲沙[51]	0（0）	53.5	7.2	3.8	13.8
靶向药物联合					
度维利塞联合罗米地辛[52]	5（5.1）	56.0	12.0	中位EFS期：3.5个月	12.0
西达本胺联合阿扎胞苷±GemOx[53]	1（3.3）	53.3（总人群） 81.3（联合化疗）	14.2	7.1（总人群），14.7（联合化疗）	8.7（总人群），38.8（联合化疗）
罗米地辛联合阿扎胞苷[54]	1（4.0）	54.0	13.5	8.0	20.6

**注** PTCL：外周T细胞淋巴瘤；ICE：异环磷酰胺+卡铂+依托泊苷；GemOx：吉西他滨+奥沙利铂；PTCL-NOS：外周T细胞淋巴瘤，非特指型；AITL：血管免疫母细胞性T细胞淋巴瘤；ALCL：间变性大细胞淋巴瘤；ORR：客观缓解率；DOR：缓解持续时间；PFS：无进展生存；OS：总生存；NA：无数据；EFS：无事件生存

（1）HDACI：西达本胺是苯酰胺类HDAC亚型选择性抑制剂，已获批用于既往至少接受过一次全身化疗的R/R PTCL患者。我国Ⅱ期单臂队列研究纳入83例R/R PTCL，包括PTCL-NOS（34％）、ALCL（22％）、AITL（13％）等。西达本胺治疗后ORR达到28％，中位PFS期、OS期和缓解持续时间（DOR）分别为2.1个月、21.4个月和9.9个月，治疗后3个月持续缓解率为24％。不同亚型之间缓解率存在差异，其中AITL、ALK^-^ ALCL、ALK^+^ALCL（或未知）、PTCL-NOS患者的ORR分别为50％、45％、33％和22％[43]。西达本胺联合化疗可进一步提高R/R PTCL患者的ORR，一项中国Ⅱ期研究显示，西达本胺联合泼尼松、环磷酰胺和沙利度胺治疗无法耐受标准化疗的R/R PTCL患者，ORR可达到71.1％[48]。最新公布的一项我国Ⅱ期多中心研究显示，R/R PTCL患者应用西达本胺联合阿扎胞苷±GemOx的ORR为53.3％，联合化疗组的ORR显著高于单药组，分别为56.3％和21.4％，且联合化疗组的PFS和OS均较单药组显著延长[53]。小样本研究显示，难治复发滤泡辅助性T细胞淋巴瘤尤其是AITL患者应用HDACI联合去甲基化药物的双表观靶向治疗的疗效较好，ORR为80％，CR率为67％[54]。

（2）靶向CD30的ADC：BV靶向CD30已获批用于R/R系统性ALCL。一项Ⅱ期多中心队列研究纳入58例患者，包括16例ALK^+^ALCL和42例ALK^-^ ALCL患者，ORR达到86％（CR率为57％），中位DOR和PFS期分别为12.6个月和13.3个月，中位OS期尚未达到。3级以上不良反应包括中性粒细胞减少（21％）、血小板减少（14％）和周围神经病变（12％）[55]。联合治疗的小样本回顾性研究显示，6例CD30阳性R/R PTCL患者接受BV联合ICE方案的ORR为66.7％[49]。

（3）蒽环类药物：米托蒽醌脂质体已获批用于R/R PTCL。最新的中国Ⅱ期单臂研究中，108例R/R成熟T细胞和NK细胞淋巴瘤患者接受米托蒽醌脂质体治疗，研究者评估的ORR为46.3％，13.9％的患者达到CR；中位PFS期和OS期分别为8.5个月和23.3个月，对既往使用过蒽环类药物治疗的患者依然有效[44]。一项真实世界研究（MOMENT，ChiCTR2200062067）纳入R/R患者188例，27例接受单药治疗，161例联合其他药物治疗，CR率为23.9％，ORR为67.6％（AITL 66.7％，PTCL-NOS 67.3％，ALK^-^ ALCL 75％，ALK^+^ALCL 75％）[56]。常见的3级以上不良反应是血液学不良反应（白细胞降低占66.7％，贫血占18.2％，血小板降低占14.1％），色素沉着、心脏不良反应及输液相关反应也需重点关注[57]。

（4）叶酸类似物：普拉曲沙是第一个被批准用于治疗R/R PTCL的叶酸类似物。一项国际多中心单臂Ⅱ期研究纳入115例R/R PTCL，普拉曲沙治疗的ORR为29.0％，CR率和PR率分别为11％和18％；中位PFS期、OS期和DOR分别为3.5个月、14.5个月和10.1个月[45]。另一项国内多中心单臂研究显示，R/R PTCL患者的ORR为52％，中位DOR为8.7个月，84％的患者在第1个疗程即可观察到首次缓解，中位PFS期和中位OS期分别为4.8个月和18.0个月[58]。Ⅰ/Ⅱ期临床研究汇总分析显示，普拉曲沙联合罗米地辛的ORR为53.5％，疾病控制率为67.8％[51]。口腔黏膜炎是普拉曲沙常见的非血液学不良反应，预防性使用亚叶酸钙可有效降低黏膜炎的发生率[59]。

（5）JAK抑制剂：戈利昔替尼是一种JAK1高选择性抑制剂，已获批用于既往至少接受过一次系统治疗的R/R PTCL患者。一项国际多中心Ⅱ期研究（JACKPOT8 Part B）纳入包括中国在内的49个中心104例既往接受过≥1次系统治疗的成人R/R PTCL，戈利昔替尼治疗后的ORR为44.3％，CR率为23.9％，中位随访12.5个月，中位DOR为20.7个月，预计中位OS期为19.4个月[42]。亚组分析显示，既往接受≥2种系统治疗和HDACI治疗患者的ORR分别为51.6％和54.5％；AITL、PTCL-NOS、其他病理亚型及ALCL的ORR分别为56.3％、46.0％、44.4％和10.0％[42]。戈利昔替尼的主要不良反应是血液学不良反应。

（6）磷脂酰肌醇3激酶（PI3K）抑制剂：林普利塞是PI3Kδ的高度选择性小分子抑制剂。一项中国Ⅱ期研究纳入88例R/R PTCL患者，林普利塞治疗的ORR为48.0％，其中CR率为30％，中位PFS期和OS期分别为5.5个月和14.2个月，6个月OS率为75％。度维利塞选择性抑制PI3K-δ和PI3K-γ，联合罗米地辛治疗R/R PTCL的ORR可达到56.0％，且在AITL/PTCL-滤泡辅助T细胞亚型中疗效更佳，ORR可达到71％[54]。PI3K抑制剂可能影响B细胞和T细胞功能导致免疫抑制，增加机会性感染（如肺孢子菌肺炎）的发生风险，故治疗期间若CD4^+^T细胞<200/µl，应常规接受复方磺胺甲噁唑预防感染。

（7）烷化剂：苯达莫司汀是一种双功能基烷化剂。苯达莫司汀单药（Ⅱ期BENTLY研究[60]）或联合卡铂和地塞米松（Ⅱ期BENCART研究[61]）的ORR分别为50％和54％，CR率分别为29％和28％，中位PFS期分别为3.6个月和4.4个月。

（8）去甲基化药物：阿扎胞苷属于嘧啶核苷类似物，能抑制DNA甲基转移酶，逆转肿瘤抑制基因的异常甲基化。一项国际多中心随机对照研究对比口服阿扎胞苷单药与其他药物（吉西他滨/苯达莫司汀/罗米地辛）治疗R/R nTFHL的疗效，中位随访27.4个月，阿扎胞苷组与对照组的中位PFS期分别为5.6个月和2.8个月，且阿扎胞苷组3级以上不良反应发生率更低[62]。

（9）免疫调节药物：来那度胺是一种免疫调节药物。一项Ⅱ期研究显示，R/R PTCL患者接受来那度胺单药治疗后的ORR为22％，中位PFS期和中位DOR分别为2.5个月和3.6个月；AITL患者的获益更为明显，ORR可达到31％[63]。

（10）ALK抑制剂：克唑替尼（Crizotinib）是ALK抑制剂，适合对BV耐药的ALK^+^ALCL患者[5]。克唑替尼联合化疗治疗R/R ALK^+^系统性ALCL的ORR为81.3％。克唑替尼联合化疗与单纯化疗的2年PFS率分别为68.7％和45％，OS率分别为86.1％和78.9％[50]。

（11）EZH1/2抑制剂：EZH1/2抑制剂HH285治疗R/R PTCL的国内多中心临床研究显示其具有显著的抗肿瘤活性，ORR为67.6％，CR率为29.4％，DOR为14.8个月，AITL亚型患者临床获益更明显（ORR为86.7％）[64]。

3. R/R PTCL的巩固治疗：对于前线治疗未进行auto-HSCT的R/R PTCL患者，治疗后疗效达到PR以上建议选择auto-HSCT巩固治疗[5]。对于auto-HSCT后复发的PTCL患者，allo-HSCT是此类患者挽救治疗后疗效达到PR以上时需优先考虑的巩固治疗手段；对于化疗耐药的R/R PTCL患者，allo-HSCT是重要的挽救性治疗手段[65]。对于年轻、适合强化疗且供体可及的患者，进行allo-HSCT可实现长期生存，荟萃分析提示，接受allo-HSCT治疗的5年PFS率为40％～48％，5年OS率为53％～54％[66]，但需权衡移植相关死亡率与复发风险。一项大型国际多中心回顾性研究结果显示，化疗敏感性差、高龄和体能状况降低的患者进行allo-HSCT可能出现更差的PFS和OS[67]–[68]。

不适合移植者的维持治疗目前尚不清楚，建议参加临床试验。

（三）疗效评价及随访

疗效评价和随访推荐采用2014年Lugano标准。
